# Effects of apparent temperature on daily mortality in Lisbon and Oporto, Portugal

**DOI:** 10.1186/1476-069X-9-12

**Published:** 2010-03-10

**Authors:** Sofia P Almeida, Elsa Casimiro, José Calheiros

**Affiliations:** 1 Centro de Investigação em Ciências da Saúde, Faculty of Health Sciences, University of Beira Interior, Portugal; 2 Climate Change Impacts, Adaptation and Mitigation Research Group (CC-IAM), Faculty of Science, University of Lisbon, Portugal; 3 INFOTOX - Environmental Health Consultants, Lisbon, Portugal

## Abstract

**Background:**

Evidence that elevated temperatures can lead to increased mortality is well documented, with population vulnerability being location specific. However, very few studies have been conducted that assess the effects of temperature on daily mortality in urban areas in Portugal.

**Methods:**

In this paper time-series analysis was used to model the relationship between mean apparent temperature and daily mortality during the warm season (April to September) in the two largest urban areas in Portugal: Lisbon and Oporto. We used generalized additive Poisson regression models, adjusted for day of week and season.

**Results:**

Our results show that in Lisbon, a 1°C increase in mean apparent temperature is associated with a 2.1% (95%CI: 1.6, 2.5), 2.4% (95%CI: 1.7, 3.1) and 1.7% (95%CI: 0.1, 3.4) increase in all-causes, cardiovascular, and respiratory mortality, respectively. In Oporto the increase was 1.5% (95%CI: 1.0, 1.9), 2.1% (95%CI: 1.3, 2.9) and 2.7% (95%CI: 1.2, 4.3) respectively. In both cities, this increase was greater for the group >65 years.

**Conclusion:**

Even without extremes in apparent temperature, we observed an association between temperature and daily mortality in Portugal. Additional research is needed to allow for better assessment of vulnerability within populations in Portugal in order to develop more effective heat-related morbidity and mortality public health programs.

## Background

Climate change impacts on human health are a global concern [1]. Recent extreme weather events, such the European heatwave in 2003, have attracted renewed interest on weather-related health effects [2,3]. Since climate change will likely increase the mean temperature, as well as the frequency of heat events [4] there is an urgent need to evaluate the links between climate and human health, to better identify vulnerable populations and take preventive measures.

Previous studies have reported that days of usually low and high ambient temperatures are associated with increases in mortality and morbidity [5-9]. Some groups adopted for a time series approach, while others focus on extreme events such as heat waves and cold spells using episode analysis. In Europe, populations living in urban environments as well as the elderly have a high risk of mortality from ambient heat exposure [10]. If future populations become more urbanized and the number of elderly continues to increase, the issue of heat-related mortality will probably become more severe.

Being part of the Iberian Peninsula, Portugal has a mild Mediterranean climate. Observed climate trends in Portugal show that between 1970-2000 there was an increase in the average temperature at a rate of 0.5°C/decade, which is more than twice than what was observed for the mean world temperature [11]. Quantitative studies looking at the effects of temperature on human health in Portugal are surprisingly few [12,13]. In this paper we investigate the association between mean apparent temperature and daily mortality in Lisbon and Oporto during the warm season using modern statistical methods accounting for confounding effects of air pollution.

## Methods

### Study areas

The study areas are Greater Lisbon and Greater Oporto (hereafter referred to as Lisbon and Oporto). The former region includes Lisbon city, the capital of Portugal and immediate urban surrounding. It is located close to the estuary of the Tagus River and in proximity to the Atlantic Ocean, which confers selectivity to the climate of the city and the surrounding regions. Oporto is the second largest Portuguese city and belongs to the Oporto Metropolitan Area. Located in the north of the country, it has a north maritime climate.

Greater Lisbon has a population close to 2.1 million people with a population density of 898 inhabitants per square kilometer; Oporto has approximately 1.2 million people and a population density of 540 per square kilometer [14].

### Health and environment data

Mortality data from April to September for Lisbon (2000-2004) and Oporto (2000-2004) were obtained from the National Institute of Statistics. Daily death counts for all-causes (except external causes) (ICD-9 codes <800; ICD-10 codes A00-R99), for cardiovascular diseases (ICD-9 codes 390-459; ICD-10 codes I00-I99) and for respiratory diseases (ICD-9 codes 460-519; ICD-10 codes J00-J98) were used. Mortality data were classified in two groups: one group for all ages and another group of >65 years.

Daily meteorological variables of mean temperature and dew point temperature for Lisbon and Oporto were obtained from the National Meteorological Institute, for the period under study. For both cities, climate data from only one station was used. In the case of Lisbon the meteorological station (Geofisico) used is in the city centre. This station is considered to be the most precise station for Lisbon, and has the most reliable data series. For Oporto the airport meteorological station (Pedras Rubras) was used as there is no reliable station in the city centre.

These variables were used to calculate the mean apparent temperature which is defined as an individual's perceived air temperature given the humidity. It is calculated with the following formula [15,16]:

In our study apparent temperature is used to represent the effect of the typical temperature exposure that is commonly experienced during the warmer months.

For both study areas, daily air pollution data were obtained from the Portuguese Institute of the Environment, for all the background stations. We calculated the daily mean concentrations of particulate matter <10 μm (PM_10_) and the daily 8-hour moving average ozone (O_3_) concentrations. For the calculation of daily air pollutants exposures at least 75% of the one-hour values were available on that particular day. For both cities, data from 3 background monitoring stations was used. In our analyses we used the same day concentration of PM_10 _and O_3 _(lag 0).

### Statistical Methods

Similar to Basu et al. [17] we investigated the association between mean apparent temperature and daily mortality using generalized additive models (GAM), with a quasi-Poisson link function, in the warm period (April to September). In the models we controlled for time trend using natural cubic spline smoothing function. The variable day of the week was included as an indicator variable. The partial autocorrelation function (PACF) was used to select the degrees of freedom for time trend. Residuals of each model were examined to check whether there were discernable patterns of autocorrelation by means of residual plots and PACF plots, respectively.

Several sensitivity analyses were performed. In an exploratory analysis, several lag times were analyzed to determine which of the following had the best model fit according to the deviance: single-day lag from 0 to 3 days and multi-day average starting from lag 0 (up to 3). By lag-01 we mean the 2-day moving average of current and previous day values. In addition, the sensitivity of results to doubling and halving the degrees of freedom (*df*) in the splines were also assessed. We found that halving the *df *underestimate the heat effects. When doubling the *df *the heat effects were similar to the base model, in which the seasonal spline *df *were chosen with standard techniques. The results thus suggest that our base model spline have adequately captured long-term variation in mortality.

For the all-causes mortality, confounding effects of air pollution was examined. Each pollutant (lag 0) was added separately in the model to see if it confounded the association between apparent temperature and mortality. In order to study the interaction between apparent temperature and air pollution, each air pollutant was included in the model specification and an interactive term (pollution times apparent temperature) was examined.

Our results are reported as the percent change in mortality per 1°C increase in mean daily temperature, and associated 95% confidence intervals (CIs). All analyses were made using GAM models in R software [18].

## Results

Table 1 presents the summary statistics on the environmental variables and mortality data for Lisbon and Oporto during the warm period. Mean deaths for all mortality causes studied were greater in Lisbon than in Oporto, reflecting the fact that Lisbon is a more populated area. Cardiovascular deaths accounted for approximately 40% of all deaths in Lisbon, and 30% in Oporto, and respiratory deaths 6.5% and 8.3%, respectively. During the study period the Oporto area had higher mean concentrations for PM_10 _and O_3_. Lisbon is a warmer city with a mean temperature of about 3°C higher than in Oporto.

**Table 1:** Summary statistics of daily mortality data and environmental variables in the warm season (April to September).

City (time period)	Variables	Mean	SD	Minimum	Median	Maximum
Lisbon	All-causes all ages	53.4	9.5	27.0	53.0	101.0
(2000-2004)	All-causes, >65	40.8	8.4	18.0	40.0	92.0
	Cardiovascular all-ages	21.7	5.8	7.0	21.0	53.0
	Cardiovascular, >65	19.1	5.5	5.0	19.0	50.0
	Respiratory all-ages	3.1	2.0	0.0	3.0	12.0
	Respiratory, >65	2.8	1.9	0.0	2.0	10.0
						
	Apparent temperature (°C)	19.6	4.3	7.9	20.3	31.6
	Mean temperature (°C)	20.0	3.8	9.7	20.4	33.5
	Diurnal Temperature (°C)	8.7	2.7	0.5	8.5	16.7
	PM_10 _(μg/m^3^)	35.4	17.0	9.7	31.1	161.4
	O_3 _(μg/m^3^)	71.7	22.2	18.0	69.9	151.3
						
Oporto	All-causes all ages	34.9	6.5	20.0	34.0	64.0
(2000-2004)	All-causes, >65	25.7	5.6	10.0	25.0	49.0
	Cardiovascular all-ages	11.3	3.6	1.0	11.0	29.0
	Cardiovascular, >65	10.0	3.4	1.0	10.0	25.0
	Respiratory all-ages	2.9	1.8	0.0	3.0	10.0
	Respiratory, >65	2.6	1.6	0.0	2.0	9.0
						
	Apparent temperature (°C)	17.0	4.1	5.8	17.5	31.3
	Mean temperature (°C)	17.3	3.3	8.3	17.4	30.4
	Diurnal Temperature (°C)	7.9	3.0	1.4	7.5	18.3
	PM_10 _(μg/m^3^)	38.0	22.4	7.3	32.3	151.6
	O_3 _(μg/m^3^)	74.2	22.7	18.4	71.4	169.8

**Figure 1:**
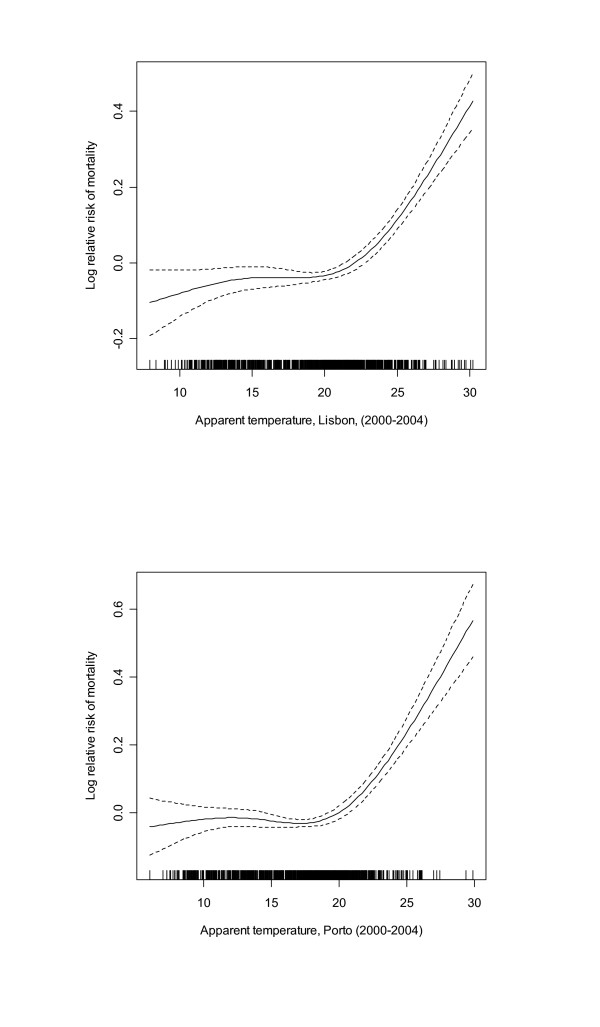
**City-specific plots of the smoothing function of mean apparent temperature in the warm season (April to September) in Lisbon and Oporto, 2000-2004**. Models in both cities adjusted for time trend and day of the week.

Figure 1 shows the associations between apparent temperature and mortality in both cities during the warm season, controlling for day of the week and long-trend trends in mortality. For both cities the mortality risk increases with increasing apparent temperature.

In the exploratory analyses, we found that lag-01 apparent temperature had the best model fit in both study areas, compared with the moving averages of multiple days. We therefore report here the results analysing the effect of apparent temperature only during warmer months and for lag-01.

In Lisbon, we found that per 1°C increase in mean daily apparent temperature a 2.1% (95%CI: 1.6, 2.5), 2.4% (95%CI: 1.7, 3.1), and 1.7% (95%CI: 0.1, 3.4) increase in all-causes, cardiovascular and respiratory mortality, respectively, for all-ages group (table 2). Overall, the elderly showed the highest effects for all-causes mortality and cardiovascular diseases during the warm season.

**Table 2:** Percentage increase (95% Confidence Interval) in daily mortality for a 1°C increase in mean apparent temperature during the warm season (April to September) in Lisbon, 2000-2004

Models^a, b^	% Increase	95% CI
All-causes mortality		
All-ages	2.1	1.6, 2.5
> 65	2.7	2.2, 3.2
Cardiovascular diseases		
All-ages	2.4	1.7, 3.1
> 65	2.8	2.1, 3.6
Respiratory diseases		
All-ages	1.7	0.1, 3.4
> 65	2.3	0.5, 4.1

^a ^Models adjusted for time trend and day of week.

^b ^*df *for seasonal natural cubic spline ranged from 22 to 24, based on partial autocorrelation function.

Table 3 shows the percent increase in mortality associated with mean apparent temperature during the warm season in Oporto. Again, we found that mortality increases with apparent temperature. A 1°C increase in mean daily apparent temperature was associated with an increase in all-causes mortality of 1.5% (95% CI: 1.0, 1.9). In contrast with the results for Lisbon, the percent increase in mortality in Oporto was highest for respiratory diseases for all-ages as well as the > 65 age group.

**Table 3:** Percentage increase (95% Confidence Interval) in daily mortality for a 1°C increase in mean apparent temperature during the warm season (April to September) in Oporto, 2000-2004

Models^a, b^	% Increase	95% CI
All-causes mortality		
All-ages	1.5	1.0, 1.9
> 65	1.8	1.2, 2.3
Cardiovascular diseases		
All-ages	2.1	1.3, 2.9
> 65	2.2	1.3, 3.0
Respiratory diseases		
All-ages	2.7	1.2, 4.3
> 65	3.0	1.4, 4.7

^a ^Models adjusted for time trend and day of week.

^b ^*df *for seasonal natural cubic spline ranged from 16 to 20, based on partial autocorrelation function.

Confounding and effect modification for each air pollutant was evaluated in separate models for all-causes mortality. In Lisbon, adding mean concentration of PM_10 _and O_3_, separately to the model with apparent temperature and day of the week, the effect decreased when adjusting for PM_10_, but did not really change when adjusting for ozone (table 4). For Oporto, the association diminished but remained positive and significant when PM_10 _and O_3 _were included in the models (table 5).

**Table 4:** Percentage increase (95% Confidence Interval) in all-causes mortality for a 1°C increase in mean apparent temperature(lag01), adjusted by individual pollutant(lag 0) in Lisbon^a^.

	% Increase	95% CI
**All-ages**		
Base model: apparent temperature + day of week	2.1	1.6, 2.5
+ Particulate matter	1.6	1.0, 2.1
+ 8-hr average ozone	1.9	1.4, 2.3
		
**> 65 years**		
Base model: apparent temperature + day of week	2.7	2.2, 3.2
+ Particulate matter	2.1	1.5, 2.6
+ 8-hr average ozone	2.5	2.0, 3.9

^a^Models adjusted for time trend and day of week.

**Table 5:** Percentage increase (95% Confidence Interval) in all-causes mortality for a 1°C increase in mean apparent temperature (lag01), adjusted by individual pollutant (lag 0) in Oporto^a^.

	% Increase	95% CI
**All-ages**		
Base model: apparent temperature + day of week	1.5	1.0, 1.9
+ Particulate matter (PM10)	1.1	0.6, 1.6
+ 8-hr average ozone	1.0	0.5, 1.5
		
**> 65 years**		
Base model: apparent temperature + day of week	1.8	1.2, 2.3
+ Particulate matter	1.4	0.8, 2.0
+ 8-hr average ozone	1.3	0.7, 1.8

^a ^Models adjusted for time trend and day of week.

## Discussion

Following the 2003 heatwave in Europe, various studies have assessed the effects of temperature extremes in various cities [2,3,19,20]. During this heatwave period, in Lisbon and Oporto excess mortalities of about 400 and 183 were reported respectively [21]. Although Lisbon was one of the few cities in Europe that had a heatwave early warning system already in place and alerts were sent out (via the media) to warn the population [22], the final analyzes of the 2003 heatwave health effect showed that the population in Lisbon (OR of 1.41) was more vulnerable than the population in Oporto (OR of 1.3) [21]. Improvements to the early warning system are currently being investigated to allow for better models and a more active response action so as to ensure more health and life gains in the future during extreme weather events [23].

Since climate change is also likely to change the average temperature, in the current study, we restricted our data to the warmer months and to a five-year period so that the estimate for mortality represents the average ambient temperature that is commonly experienced, not the worst scenario that may occur in years with unusual weather patterns, such extremes temperatures and heat waves. To our knowledge, this is the first study to analyse the effects of mean apparent temperature on mortality in any Portuguese city.

Our results show an association between mean apparent temperature and daily mortality in Lisbon and Oporto, during the warmer months. These associations persist even when controlling for the effects of air pollution. Overall, the population in Lisbon was more vulnerable than the population in Oporto. It is interesting to note that this result is in agreement with observation reported during the 2003 heatwave were Lisbon was more vulnerable to acute heatstress [21].

When analyzing by cause of death, we observed a strong temperature effect on cardiovascular mortality, in agreement with other studies [24-28]. From a public health point of view, this finding is important since cardiovascular diseases are the leading cause of death for both men and woman in Portugal, accounting for ~40% of all deaths during the study period. This increase in mortality is likely to be related to the stress placed on the respiratory and circulatory systems to increase heat loss though skin surface blood circulation [29].

Direct comparisons with previous investigations is difficult because earlier studies have used different climate variables, seasons in the year, modelling approaches, temperature cut-offs and percentiles [3,5,15]. Nevertheless, a previous study that analyzed the effect of temperature on all-cause mortality in New Delhi, Sao Paulo, and London found a 2.2%, 1.6% and 1.4% increases per degree Celsius above 20°C [7]. Although this study used temperature (not apparent temperature), the cut-off temperature in this study was near to the mean apparent temperature as well the mean observed temperature in Lisbon, and the results are similar.

More recent studies using analysis method similar to ours allow for more suitable comparisons. For example, a study in California, found a 2.3% increase in all-cause mortality per 10° F (about 5.5°C) increase in mean apparent temperature [17], while in another study in nine US cities an increase of 2.74% in all-cause mortality was found [30]. It is difficult to directly compare our results with other epidemiologic studies from European cities because of the various definitions used to classify temperature exposure and the different methodologies applied [8]. Studies in European cities typically use maximum apparent temperature [8], maximum temperature [9] or mean temperature [24] to analyze the effects of heat exposure above specific heat thresholds. Nevertheless, most of these studies yield results similar to ours in that risks for deaths from cardiovascular and respiratory diseases increase with increasing summer ambient temperatures.

Our finding that the elderly are the group most at risk in the warmer periods is also consistent with previous studies [8,10,31,32]. In our study the risk of death for people > 65 years was higher for cardiovascular diseases in Lisbon while in Oporto the risk was higher for respiratory diseases. A possible explanation for a higher risk for respiratory diseases in Oporto than in Lisbon is likely related to the higher pollution levels in Oporto. The latter city is surrounded by heavy industry, whereas Lisbon is a financial and administrative city surrounded by green areas and agriculture. The higher risk for cardiovascular disease in Lisbon can also be partly explained by the fact that this city is not only naturally warmer but it is also more prone to heat island effects since it is more urbanized and has a higher density of high rise buildings. Nevertheless, these differences in cause of death between these two locations should be explored in more detail in future investigations.

The reasons why confounding effect of air pollutants on heat related deaths differs between locations is an ongoing area of investigation. Thus, the inclusion of air pollutants to examine confounding and effect modification is an important contribution. We found a lower effect when adjusting for PM_10 _in Lisbon, and a decrease in the effect with PM_10 _and ozone in Oporto. Our results are consistent with those from O'Neill and colleagues [33] who found a small decrease in the association between mortality and apparent temperature when adding PM_10 _and ozone singly or jointly. A recent analysis of the effect of temperature in nine US cities, with similar methods to ours, also found a lower effect when adjusting for ozone [29].

The results from our study support the importance of examining subgroups in specific locations. The fact that in both cities the elderly were identified as being more sensitive to heat exposure, together with the fact that the elderly population in Portugal is increasing makes this finding an important public health concern warranting special attention in current and future health programs. Since climate change is likely to result in more frequent, intense and longer duration episodes of heat waves the need for continued vigilance of heat exposure as well as the development of better heat warning systems is paramount. The fact that in this study a lag-01 was used also allows for better planning of intervention actions. In addition, our results also highlight which populations are more vulnerable thus allowing for targeted public awareness of ways to avoid heat exposure. Public education targeting homes for the aged, health care centres, pharmacies, and associations/societies with close contact with the aged will be key at reducing population vulnerability to heat exposure in these two cities.

In this study we used mean apparent temperature to assess heat exposure. Although this index is a mean daily value, it requires that first hourly apparent temperatures be calculated from which the daily mean is then derived. Consequently, hourly climate data is always needed. Obtaining hourly climate data at the city level from future climate models is currently not very practical, thus limiting the use of this index in studies assessing climate change health impacts due to heat exposures. Heat exposures in terms of maximum daily temperature (not apparent temperature) would be better suited for studies aiming to use the exposure-response relationship in climate change investigations.

In future analysis it would also be interesting to explore the effect of temperature in daily hospital admissions. Acclimatization and individual susceptibility should also be investigated as possible explanations for the differences between the cities. Finally, the effect of access to prevention and to emergency care [34] between cities is also an area warranting future research.

## Conclusion

In conclusion, our study provides evidence of increased mortality due to mean apparent temperature exposure in Portugal, even when adjusting for air pollution. As changes in temperature due to climate change are expected to increase, the need for local epidemiologic studies which can help to analyse the effects on human health increases. Results of such studies can contribute towards decision making at national and local governmental agencies interested in developing better warning and health systems that allow them to be better equipped to prevent heat-related mortality. Since the response functions differ between locations, further research focusing on socio-economic, health systems management and geographical characteristics is needed.

## Competing interests

The authors declare that they have no competing interests.

## Authors' contributions

SPA and EC designed the study, data collection and analysis, wrote and reviewed the manuscript. SPA performed the statistical analysis. JC was involved in the initial stages of the study design and in revising the manuscript. All the authors read and approved the final manuscript.
